# Impact of Capsulotomy on Hip Biomechanics during Arthroscopy

**DOI:** 10.3390/medicina58101418

**Published:** 2022-10-09

**Authors:** Hyeonjoon Lee, Wonbong Lim, Seunghyun Lee, Sungmin Jo, Suenghwan Jo

**Affiliations:** 1 Department of Orthopaedics Surgery, School of Medicine, Chosun University Hospital, Gwangju 61453, Korea; 2 School of Medicine, Chosun University, Gwangju 61452, Korea

**Keywords:** biomechanics, capsulotomy, hip joint capsule, iliofemoral ligament, zona orbicularis

## Abstract

*Background and Objectives*: Anterior capsulotomy is routinely performed in hip arthroscopy to improve joint visualization; however, this can partly or completely disrupt the stabilizing ligaments of the hip. This study aimed to report the effects of conventional and extensive arthroscopic capsulotomies on hip stability. *Materials and Methods*: Eight freshly frozen cadaveric pelvises were used in this study. The range of motion and translation were measured and compared among different capsular conditions utilized in hip arthroscopy, with a special interest in the iliofemoral ligament (IFL) and zona orbicularis (ZO). The conditions included intact capsule, interportal capsulotomy, T-capsulotomy, complete IFL disruption, and complete IFL and ZO disruption. Internal rotation at three flexion planes (−10°, 0°, and 30°) and external rotation at six flexion planes (−10°, 0°, 30°, 60°, 90°, and 110°) were measured with corresponding femoral head translation distance at the application of 2.5 Nm torque. *Results*: As compared to an intact capsule, a significant increase in external rotation was observed after interportal capsulotomy from −10° to 60° and after T-capsulotomy from −10° to 110° flexion. A significant translation was observed only with a T-capsulotomy, which ranged from 1.9 to 2.3 mm across the flexion angles. Compared with conventional interportal capsulotomy, disruption of the entire IFL resulted in a significant increase in external rotation in all flexion planes, and significant translation was accompanied by disruption of the ZO. *Conclusions*: Interportal capsulotomy can result in an increase in range of motion, and T-capsulotomy can lead to significant translation. Partial or complete tears of the IFL and ZO can result in further external rotation and translation.

## 1. Introduction

Arthroscopy is a popular diagnostic and treatment tool for various hip joint pathologies. Compared to open surgical methods, hip arthroscopy provides advantages in patient morbidity and recovery time, and a growing number of hip arthroscopy procedures are performed to treat various hip conditions with favorable outcomes [1,2]. Unlike other joints that utilize arthroscopy, the hip is confined by rigid capsuloligamentous structures and combined with a thick pericapsular soft tissue, manipulation of arthroscopic devices within the joint is often restricted. Therefore, capsulotomy is routinely performed to aid the mobility of instruments and to enhance visualization [3]. Currently, the common capsulotomy technique involves conjoining anterolateral portal and direct anterior or mid-anterior portal with a capsular incision beginning 1 cm from the acetabular rim (interportal capsulotomy) [4,5,6]. However, when the pathologic lesion involves the peripheral compartment, as in the case of large cam-type deformity or abundant synovial chondromatosis, an extended capsulotomy may be necessary by further extending the capsulotomy distally or by creating an additional transverse incision along the femoral neck (T-capsulotomy) [4]. While routine capsulotomy is an essential step for successful technical completion of the operation, it often involves partial or complete disruption of the static stabilizers, such as iliofemoral ligament (IFL) or zona orbicularis (ZO). IFL is a thick ligament that runs from the anterior inferior iliac spine to the lesser trochanter and ZO is a circular fiber of the capsule encircling the femoral neck [7,8]. As both structures are known to play an important role in hip stability, possible instability following capsulotomy procedures has become a concern [7,9,10,11,12].

Previous cadaveric studies have explored the effect of disrupting ligaments around the hip and reported an increase in external rotation (ER) and extension when the IFL was removed [9,11,12]. Moreover, several clinical case studies have reported the incidence of instability following hip arthroscopy and suggested that an excessive capsulotomy or redundancy of the capsule could potentially result in micro-instability or even dislocation [13,14,15]. However, studies that explored the change in hip biomechanics when stabilizing ligaments are violated by capsulotomy are scarce [11,12]. As the recent literature has cautioned against excessive capsulotomy and proposed the necessity of performing capsule repair to restore the normal anatomy of the hip capsule, validating the effect of different capsulotomies on hip biomechanics is necessary [4,16].

This study aimed to determine the effect of capsulotomy on the stability of the hip joint, with a special focus on the disruption of the IFL and ZO. To achieve our goal, we compared the range of motion and translation of the femoral head under different capsulotomy conditions at multiple flexion planes. We hypothesized that the extent of capsulotomy would correlate with an increase in range of motion and translation.

## 2. Materials and Methods

### 2.1. Specimen Preparation and Test Setup

**Figure 1:**
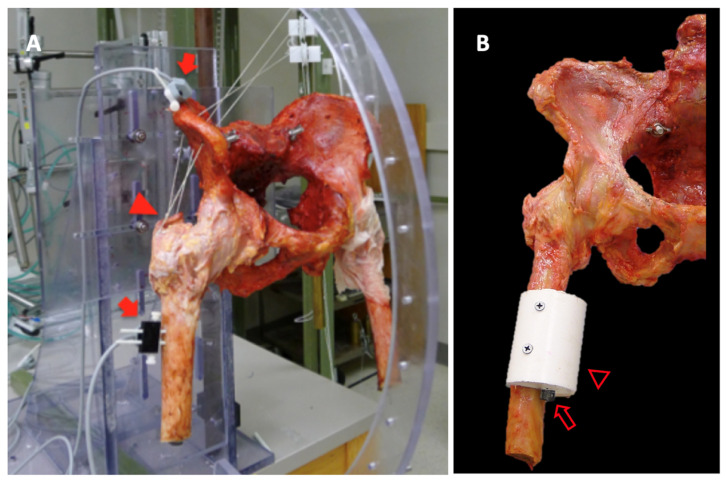
(**A**). Entire pelvic specimen was used for the test with electromagnetic sensors attached to the iliac spine and femur (arrow). The sutures loaded with 18 N weight were attached to the abductor tendons (arrowhead) to maintain a compressive force to keep the femoral head in contact with the acetabulum. (**B**). Adopter (hollow arrowhead) with a screw head (hollow arrow) was attached to the femur. Using the screw head, torque could be applied along the mechanical axis of the femur.

Fresh frozen human pelvis specimens were initially screened using fluoroscopy, and eight specimens (from three males and five females) that did not indicate evidence of osteoarthritis or abnormal bony morphology, such as dysplasia or acetabular over-coverage, were selected. The mean age of cadavers was 72 years, which ranged from 59 to 78. All musculature and soft tissues were dissected, leaving only capsuloligamentous structures intact. The proximal femur was transected approximately 16 cm distal to the lesser trochanter, and the reference points for the medial and lateral epicondyles were marked at the distal end of the stump. The pelvis was mounted on a custom-built testing apparatus to secure the specimen in an upright position. The sutures were attached to the abductor tendons loaded with weight to apply an 18-N compressive force to maintain contact of the femoral head with the acetabulum [17] (Figure 1A). The entire pelvis was used to accurately define the orientation of the pelvis; however, only one side of the hip (right) per pelvis was tested to eliminate the bias from testing structurally similar paired hips.

The electromagnetic tracking system (Polhemus, Colchester, VT, USA), which has a static accuracy of 0.76 mm and an angular accuracy of 0.15°, was fixed to the iliac crest and femoral shaft to measure the joint motion. Anatomic co-ordinate systems were defined according to the International Society of Biomechanics (ISB) guidelines using two anterosuperior iliac spines (ASIS) and two posterosuperior iliac spines (PSIS) as reference points [18]. The hip center of rotation was estimated using a functional approach that was achieved by performing flexion/extension, abduction/adduction, and circumduction three times while monitoring the 3D relative movement between the femur and pelvis [19,20]. The neutral position was defined as a position when the femoral shaft aligned with the sagittal plane comprising ASIS and pubic symphysis, and the neutral axial rotation was defined according to the previously marked points for femoral epicondyles [21].

### 2.2. Test Process

**Figure 2:**
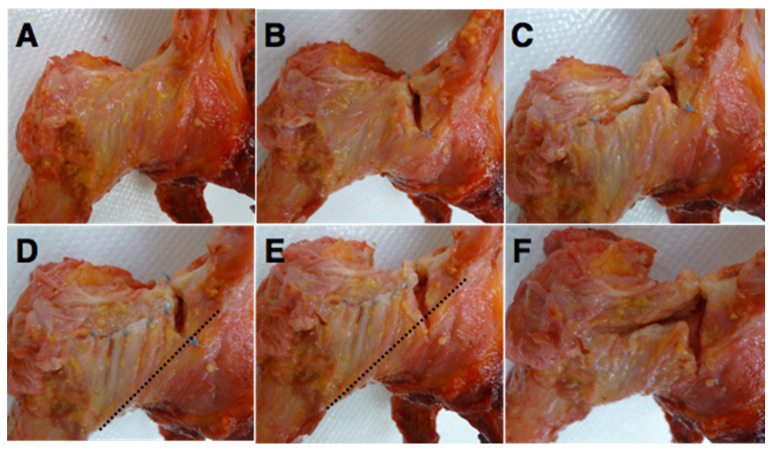
The condition of the capsule tested. (**A**). Intact capsule. (**B**). Interportal capsulotomy. (**C**). T-capsulotomy. (**D**). Repair to interportal capsulotomy. (**E**). Entire iliofemoral ligament (IFL) resection. (**F**). IFL and zona orbicularis (ZO) resection. The dotted line indicates the lower margin of IFL.

The range of motion and translation were measured under the following six conditions, which were performed sequentially as follows: intact capsulotomy, conventional interportal capsulotomy, conventional T-capsulotomy, repair to interportal capsulotomy, entire IFL resection, and entire IFL and ZO resection (Figure 2). To create interportal capsulotomy, an incision was made from the 12:30 to 2:30 position, 1 cm lateral to the acetabular rim, which resulted in an approximately 3.0-cm capsular cut [4,22]. IFL fiber on the anterior capsule was identified so that portion of the IFL is preserved. A single stitch was added at the proximal and distal ends of the incision with a 2-0 FiberWire (Arthrex, Naples, FL, USA) to prevent further extension of the incised capsule during the test. A T-capsulotomy was simulated by creating an additional incision along the femoral neck. For the purpose of the current study, ZO was resected in the T-capsulotomy and this was performed by making an incision approximately 3.5 cm in length. Following T-capsulotomy, the transverse incision was repaired to recreate an interportal capsulotomy condition using four simple stitches with a size 2 FiberWire, approximately 2 mm from each margin of the incised capsule [23]. The entire resection of IFL was performed by extending the previous horizontal cut distally along the labrum to a 3:30 or 4:00 position, which resulted in an overall incision length of 4.0 cm [24]. An entire IFL and ZO resection was simulated by removing the stitches that had been added to the transverse incision. All tests were performed at room temperature, and a saline solution was sprayed every 5 min to keep the specimen moist. During the test, each end of the incised capsule was monitored to check if any additional tearing of the capsule occurred during torque application. 

The test started with measuring flexion and extension of the hip with the application of 2.5 Nm torque. Hip ER and internal rotation (IR) were simulated by applying a torque force along the mechanical axis of the hip. To achieve this, a cement adopter with a hexagonal screw head was attached at 4–8 cm distal to the lesser trochanter, depending on the original height of the specimen. The axis of the adopter was arranged such that the torque could be applied at 6° varus from the anatomical axis of the femur (Figure 1B). A 2.5 Nm torque was applied manually using a custom-made screwdriver by rotating the hexagonal screw head in the adopter. 

The ER and IR of each capsular condition were measured at 10° extension and at 0°, and at 30° flexion. Considering function of the ligaments, only ER was measured at 60°, 90°, and 110° of flexion. The position of the femoral head’s center of rotation was monitored simultaneously when the hip was axially rotated, and the x, y, and z co-ordinates at 2.5 Nm were compared. The x, y, and z co-ordinates in the current study represent the medial/lateral, anterior/posterior, and superior/inferior directions, respectively.

### 2.3. Statistical Analysis

Two parameters were compared according to different capsular conditions as follows: range of motion for ER, IR, flexion, and extension and co-ordination of the femoral head center of rotation at the maximum range of motion. The translation was calculated by subtracting coordinates of the femoral head center of rotation at IR from ER when 2.5 Nm was applied, respectively. Therefore, the translation result was available for the hip at 10° extension and at 0° and 30° flexion. The primary outcomes were measured in degrees for a range of motion and millimeters for displacement. A repeated-measures analysis of variance was performed independently for ER, IR, flexion, extension, and translation for each hip flexion plane for the capsule of interest using the JMP software (SAS Institute, Cary, NC, USA). Mauchly’s test of sphericity was used to assume sphericity and, when the result was significant, an additional Huynh–Feldt correction was applied. For the conditions that demonstrated significant differences, a pairwise repeated-measures analysis was performed with a Bonferroni correction of the α value. For all analyses, the significance was set at *p* = 0.05. A sample size of eight was calculated with an effect size of 40% and an estimated standard deviation of 50% of the maximal axial rotation value with 80% power.

## 3. Results

### 3.1. Effect of Conventional Capsulotomy

**Figure 3:**
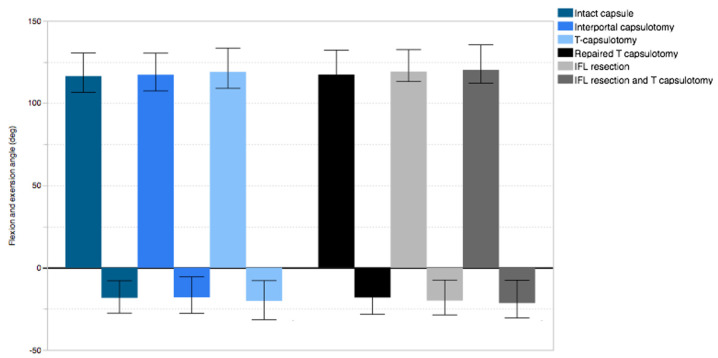
Flexion and extension angle in different capsule conditions.

The mean flexion was 117.1° ± 7.7° in intact capsule and was measured as 117.5° ± 7.2° and 118.1° ± 7.4°, respectively, for interportal capsulotomy and for T-capsulotomy. No significant differences were observed among the three capsular conditions (*p* = 0.222). The mean extension for the intact capsule was 18.3° ± 7.3° and indicated a significant increase for interportal capsulotomy (1.7° ± 1.3°, *p* = 0.008) and T-capsulotomy (3.1° ± 2.8°, *p* = 0.016) (Figure 3). 

**Figure 4:**
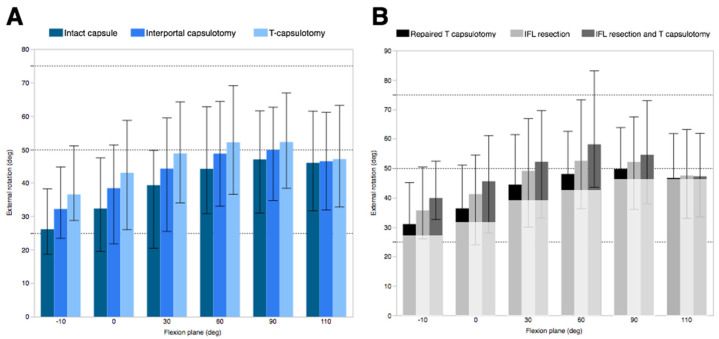
External rotation measured at different flexion angles to compare (**A**) conventional capsulotomy and (**B**) different IFL and ZO conditions. The lighter-colored box in panel B indicates the external rotation of the intact condition.

**Figure 5:**
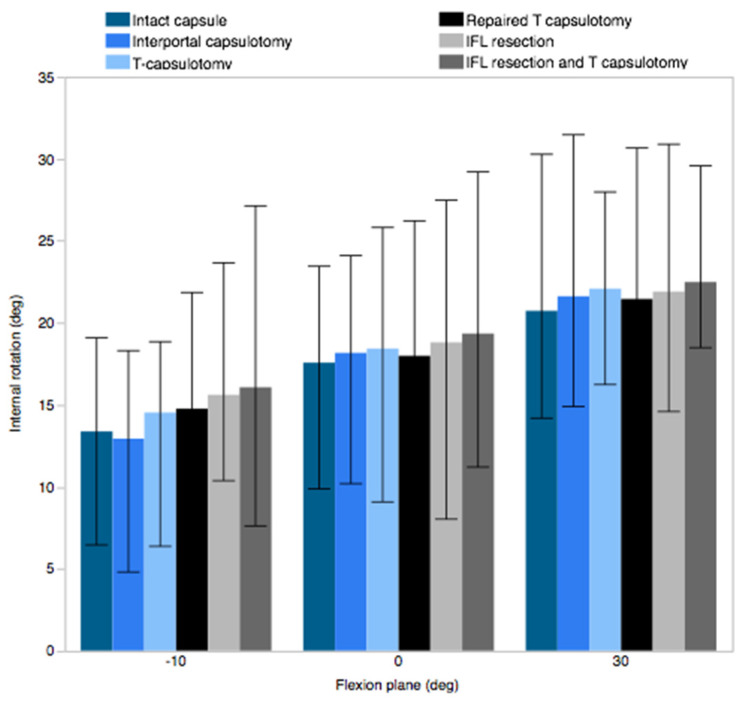
Internal rotation measured at −10°, 0°, and 30° flexion.

The ER observed in the specimens with an intact capsule increased with a flexion angle up to 90°, ranging from a mean of 26.2° ± 7.7° at 10° extension to 47.0° ± 11.2° at 90° flexion. Compared to the intact capsule, interportal capsulotomy resulted in a significant increase in ER up to 60° flexion, while T-capsulotomy demonstrated a significant increase up to 110° flexion. The increase in the ER was most pronounced at 10° of hyperextension position, where ER increased by 6.1° ± 3.4° after interportal capsulotomy and 10.4° ± 6.4° after T-capsulotomy (Figure 4A). However, the difference in IR was negligible and did not reach significance across different capsular conditions (Figure 5).

**Figure 6:**
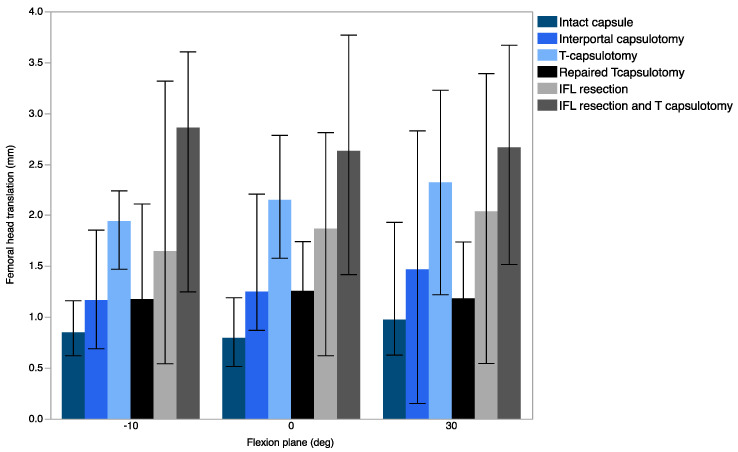
Translation measured as a change in femoral head center of rotation in −10°, 0°, and 30° flexion.

Translation occurred mainly in the anterolateral direction, while translation in the superior/inferior direction was negligible. Across the flexion planes tested, no significant differences in translation were observed between the intact capsule and interportal capsulotomy; however, a significant increase was noted when compared with T-capsulotomy. The difference was the greatest in 30° flexion with the mean translation of 1.0 mm ± 0.5 mm (Figure 6).

### 3.2. Effect of IFL Disruption

After the repair of the transverse incision to recreate interportal capsulotomy, an additional incision distally to disrupt the entire IFL resulted in a significant increase in ER in all flexion planes, ranging from 0.8° ± 0.6° (110° flexion) to 5.9° ± 4.3° (0° flexion) (Figure 4B). However, no difference was observed in translation across the flexion planes (Figure 6).

T-capsulotomy was compared with the entire IFL and ZO resection conditions to determine the effect of IFL resection in the presence of ZO disruption (Figure 2C,F). The entire IFL resection with the presence of ZO disruption resulted in significantly increased ER in up to 60° flexion and greater translation at 10° extension (0.9 mm ± 0.8 mm). The greatest ER was observed at 60° flexion (6.0° ± 6.3°). The flexion, extension, and IR remained insignificant in all comparisons.

### 3.3. Effect of IFL and ZO Disruption

When interportal capsulotomy was compared with T-capsulotomy (Figure 2B,C), the resection of ZO resulted in a significant increase in ER up to 90° flexion, and the difference was most pronounced at 30° flexion (4.5° ± 3.6°). Additionally, significant translation was observed at −10° extension (*p* = 0.0052) and at 0° flexion (*p* = 0.0050), which was 1.2 mm ± 0.4 mm and 1.0 mm ± 0.1 mm, respectively. No significant differences were observed in IR, flexion, or extension.

When the entire IFL was resected (Figure 2E,F), additional ZO resection resulted in a significant increase in ER up to 60° flexion, with the greatest difference at 60° flexion (6.0° ± 6.3°) (Figure 4B). Significant translation was observed in 10° extension (*p* = 0.0490) and in 0° flexion (*p* = 0.0262), with 1.2 mm ± 1.1 mm and 0.8 mm ± 0.6 mm translation, respectively. Translation data of femoral head are summarized in Table 1.

## 4. Discussion

The recent advancements in hip arthroscopy are largely attributable to capsulotomy, which enables easy and safe access to key pathologic lesions in the central and peripheral compartments [3,4,5,25]. Capsulotomy is performed in the upper anterior quadrant, which inevitably causes partial disruption to the IFL; however, the extent of capsulotomy varies among surgeons. As incomplete correction of pathologic lesions has been implicated as the main reason for failed arthroscopy, performing extensive capsulotomy, which may require further extension through the entire IFL or ZO according to the location and extent of the lesion, is often essential [26,27]. However, postoperative dislocation or subluxation after a hip arthroscopy procedure has been reported, suggesting that instability could develop due to an insufficient capsule [13,14,15]. However, the effect of capsulotomy has not been described in detail, and the rationale for repairing the capsule during hip arthroscopy remains unclear.

The capsule is composed of inner capsular components and outer ligamentous structures and has been considered an important static stabilizer that limits excessive range of motion. The anterosuperior portion of the capsule is covered largely by the inverted Y-shaped IFL, which originates from the anterior inferior iliac spine and plays a significant role in limiting ER and extension. Disruption of this structure could result in excessive movement and potential instability [9,11,12,28,29,30]. 

**Figure 7:**
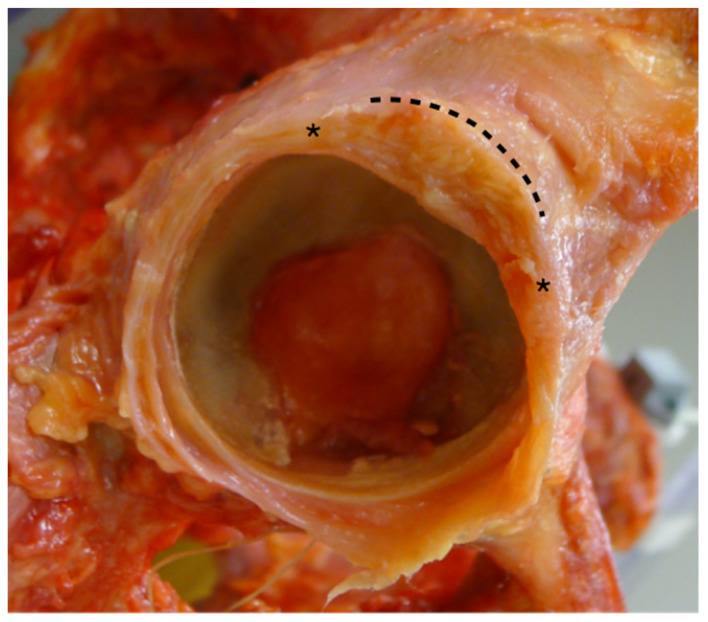
The thickest portion of the anterior capsule, which includes IFL, locates in the anterosuperior region, as marked by asterisk and dotted line.

The influence of the IFL on hip stability has been well documented. The study from Myers et al. assessed the role of the labrum and IFL and reported increases of 12.9° in ER after the IFL resection [12]. Martin et al. have reported an increase of 7.0° and 16.7° when the medial and additional lateral IFL was resected sequentially when the hip was in neutral flexion [11]. The results of our study also demonstrated that the ER increases with the extent of IFL disruption. The ER increased up to 10.4° when the IFL was resected, although it was also demonstrated to increase, albeit slightly when only a small portion of it was disrupted, as can be observed in a conventional interportal capsulotomy. The degree of difference as compared to the intact capsule was the greatest at 10° of extension. This is consistent with those reported previous studies in that, because of the orientation of the ligament, damage to the IFL could result in instability in extension and ER [4,7,12,28]. However, our study revealed that, even in the more flexed positions, when the IFL is relatively lax, ER consistently demonstrated a significant increase. We also noticed that, while the IFL lies broadly in the anterior capsule, the capsule was the thickest in the anterosuperior quadrant (Figure 7). Therefore, even with a small capsulotomy, increases in the range of motion may be unavoidable. However, femoral head translation was negligible in interportal capsulotomy, indicating that micro-instability is not a significant problem in small capsulotomy.

Another finding in the present study was the significant effect of ZO on hip stability. As ZO is located close to the head–neck junction of the femur, conventional T-capsulotomy can result in disruption of the ZO. The anatomic feature and function of ZO are poorly understood and often described as a locking ring around the femoral neck, which plays a significant role in preventing distraction [4,7]. Our study revealed that the resection of ZO resulted in a further increase in ER and significant translation. Considering the shape and location of the ZO, the ZO may prevent translation by acting as a physical barrier, thus possibly explaining the negligible translation when ZO was not disrupted. Moreover, when the IFL and ZO were both incised, both translation and ER were the greatest. As the capsule and surrounding ligamentous structures may tighten the hip joint in a screw-home mechanism, the disruption of these ligaments may violate this mechanism and result in instability.

We attempted to minimize bias using the following method. First, we used the entire pelvis instead of the hemipelvis as the current guidelines from the ISB for measuring pelvic orientation involve specific landmarks, including bilateral ASIS and PSIS. Second, only one hip per pelvis was considered. The anatomical difference between the bilateral hips has been reported to be small and the results would likely be similar. Our pilot study revealed a <5% difference in the range of motion between the hips of the same specimen; therefore, we decided to use only one hip per pelvis to minimize bias resulting from testing similar structures.

However, this study had several limitations. First, the accuracy of the center of rotation is debatable. Following the recommendation of ISB, we opted to use the functional approach, as it has been validated as the most accurate method for determining the center of rotation [18,20]. However, even a slight offset of the center of rotation could result in measuring the displacement from rotation rather than translation. To the best of our knowledge, only one study has reported the effect of capsulotomy on translation. Myers et al. measured the anteroposterior translation of the femoral head during ER and reported a −0.4 ± 0.1 mm posterior translation in an intact capsule, which increased to 1.4 ± 0.5 mm after IFL resection [12]. Our results demonstrated more translation, which is likely due to considering displacement in three planes. Second, the specimen characteristics may have influenced the results. The donors of our study were older than typical patients who receive hip arthroscopy surgery; therefore, the capsule might be stiffer. However, acquiring cadavers of young age is extremely difficult, and this remains as a limitation of the study. Finally, this study did not consider the effect of muscles around the hip or instability caused by venting the capsule. Therefore, the results of the current study may be different in vivo. We believe a study design utilizing loaded translation machines with load cells would provide better information on the stabilizing role of ligaments in the joint. Additionally, the clinical relevance of the increases in range of motion and translation observed in the current study is unclear. Biomechanical studies on actual surgery should be interpreted with extreme caution as it is only suggestive results at time zero, and the outcomes after spontaneous healing of the capsule are unknown. 

Nevertheless, our study demonstrated that hip ER and translation of the femoral head were influenced by the extent of capsulotomy performed. Conventional interportal capsulotomy, disrupting only the portion of the IFL, resulted in an increase in ER, while an additional transverse incision resulted in anterolateral translation. Resecting both IFL and ZO contributes to an increase in ER, while ZO may play a more critical role in preventing translation. Hence, capsule repair would be beneficial following extensive capsulotomy. 

## 5. Conclusions

Interportal capsulotomy can result in an increase in the range of motion, and T-capsulotomy can lead to significant translation when ZO is violated. The complete disruption of both IFL and ZO can result in further ER and translation. 

## Figures and Tables

**Table 1:** Translation of the femoral head center of rotation as calculated by average root mean square of the difference of co-ordinate between external rotation and internal rotation at 2.5 Nm torque. Units in mm.

	10° Extension	0° Flexion	30° Flexion
Intact capsule	0.8 ± 0.2	0.8 ± 0.2	1.0 ± 0.5
Interportal capsulotomy	1.2 ± 0.4	1.0 ± 0.1	1.5 ± 0.9
T-capsulotomy	1.9 ± 0.3	2.1 ± 0.5	2.3 ± 0.8
Reproduced interportal capsulotomy	1.2 ± 0.6	1.3 ± 0.4	1.2 ± 0.5
Extended interportal capsulotomy	1.6 ± 0.9	1.9 ± 0.8	2.0 ± 1.1
Extended T-capsulotomy	2.9 ± 0.9	2.6 ± 1.0	2.7 ± 0.9

## Data Availability

The data can be made available upon request.
